# The Impact of Global Health Experiences on the Emergency Medicine Residency Milestones

**DOI:** 10.1177/23821205221083755

**Published:** 2022-05-11

**Authors:** Alison Schroth Hayward, Sean S. Lee, Katherine Douglass, Gabrielle A. Jacquet, James Hudspeth, Jessica Walrath, Bradley A. Dreifuss, Janette Baird, Janis P. Tupesis

**Affiliations:** 1 Department of Emergency Medicine, Brown University Warren Alpert School of Medicine, Providence, RI, USA; 2 Department of Emergency Medicine, The George Washington University, Washington, DC, USA; 3 Department of Emergency Medicine, Boston Medical Center, Boston, MA, USA; 4 Department of Emergency Medicine, Yale Medical School, New Haven, CT, USA; 5 Department of Emergency Medicine, University of Arizona Colleges of Medicine and Public Health, Tucson, AZ, USA; 6 Department of Emergency Medicine, University of Wisconsin - Madison, Madison, WI, USA

**Keywords:** global health, milestones, emergency medicine, graduate medical education, competency-based education

## Abstract

**OBJECTIVES:**

Identify the impact of experiences in global health (GH) on the Accreditation Council for Graduate Medical Education (ACGME) competencies in emergency medicine (EM) residents and describe the individual characteristics of EM residents with global health experience compared to those without.

**METHODS:**

From 2015 to 2018, 117 residents from 13 nationally accredited United States EM residency training programs were surveyed. Specifically, the survey gathered demographic data and information regarding timing, type, location and duration of short term experiences in global health (STEGH). The survey collected both qualitative and quantitative data regarding resident experiences, including number of procedures performed and self-assessment of the impact on their residency milestones. ACGME milestone data from survey respondents was collected from each resident's training program coordinators. Chi-squared analysis and t-tests were conducted to assess differences between residents with STEGH and those without. A generalized linear model (GLM) was utilized to assess the effects of time and experience with interaction on achieving milestones in each of the competency domains, to compare milestone achievement over time between residents with STEGH and those without.

**RESULTS:**

Out of 117 EM residents, 60 were female (44%), the mean age was 30 years (standard deviation = 3.1), and 84 (71.8%) reported STEGH in general, including prior to residency (64.5%). 33 (28.2%) reported having completed STEGH during residency. The results of the GLM analysis showed that residents with STEGH during residency had significantly higher scores compared to those without the experience or STEGH pre-residency across all six competencies

**CONCLUSIONS:**

STEGH in EM residents was associated with higher milestone achievement in certain ACGME competency domains including medical knowledge, practice-based learning and improvement, and professionalism. Participation in STEGH during residency appeared to show the strongest effect, with higher scores across all six competencies.

## Introduction

Global health experiences (GHE) are becoming increasingly more common among physicians-in-training.^1^ Specifically, a growing body of academic work has developed around short term experiences in global health, currently also known in the global health literature by the acronym STEGH, and recent publications have focused on how to formalize and optimize these experiences in the expanding field of available opportunities.^2–4^ Over 30% of medical students report having had some STEGH, and up to 90% of residency programs, including approximately 75% of emergency medicine (EM) programs, offer international elective rotations.^5–8^ National surveys of residency applicants have shown STEGH to be viewed as a positive attribute of a residency program, underscoring the increasing demand.^9^

The ACGME has defined 6 general competency domains for residency training. These competency domains are patient care; medical knowledge; practice-based learning and improvement; interpersonal and communication skills; professionalism; and systems-based practice. Each competency is comprised of different milestones which residents are required to master at key stages of their medical training. The specialty of EM has its own set of these “competency-specific developmental outcomes (eg, knowledge, skills, attitudes, and performance), which represent significant points in development for learners and can be demonstrated progressively by residents from the beginning of their education through graduation to the unsupervised practice of their specialties”.^10^ It has been posited by GH-focused educators that the GH electives offered in EM residency programs may complement the Accreditation Council for Graduate Medical Education (ACGME) curriculum, helping residents to progress towards their milestones while on their electives.^11^ In addition, a novel set of competencies are being created to measure trainees’ acquisition of a fund of knowledge and skills specific to STEGH, as these electives can have marked variability in their structure and quality.^2,12^

Despite widespread participation in STEGH during residency training, the varied nature of these electives, ranging from clinical work to research, creates challenges in studying their effects on residents. Studies on the effects of STEGH in residency training have shown moderate association in promoting career choices focused on caring for the underserved populations.^13,14^ However, there is limited evidence of other outcomes associated with such experiences, such as whether the skills acquired during the STEGH directly impact the ACGME competencies.^15^ Our study attempts to determine the impact in each area of ACGME competency, using resident milestone ratings, as well as to assess individual characteristics of EM residents with GH experience compared to those without. Understanding the extent to which these electives affect milestone achievement will help to assess the utility of global health experience in applicants to residency programs, as well as STEGH in residency training.

## Methods

Human Subjects Committee Review: This study was reviewed and approved as exempt by the IRB at Yale University as an evaluation of the quality of current educational programs.

Study population: The study population consisted of residents in selected United States-based EM residency programs between 2015 and 2018. The programs from which residents participated in the study, and the numbers of respondents at each program, are listed in Table 1.

**Table 1.** Participating emergency medicine residency programs.

Program Name	Number of Respondents	Number of Residents
Boston Medical Center	18	48
Brigham and Women's/HAEMR	6	64
Brown University	8	52
Denver Health	8	68
Detroit (Detroit Receiving/Sinai Grace)*	18	42
George Washington University	5	40
Palmetto Health	9	39
Rutgers University	3	36
UH Cleveland*	3	30
University of Arizona	4	18
University of Massachusetts	4	36
University of North Carolina	1	30
University of Wisconsin	17	36
Yale University	15	60

*Sites from which we could not obtain milestone data of respondents. Data was received from one of two sites in Detroit.

Data collection and study design: Residents at participating EM residency programs received an invitation to take an online survey via an e-mailed recruitment message. Respondents were offered the opportunity to opt in to a drawing for a $50 iTunes gift card for their participation. Residents were invited to take this survey whether they had participated in previous STEGH or not. Each resident was individually consented to participate. The survey included consent for the study team to contact the resident's program coordinator to request their milestone data, as well as their demographic information, information regarding timing, type, location and duration of the GH experience. The survey also collected both qualitative and quantitative data regarding resident experiences, including the number and types of procedures performed and self-assessment of the impact of their experience on their residency milestones. Demographic data of the respondents is represented in Table 2.

**Table 2.** Baseline characteristics of EM resident survey respondents.

	All residents (n = 117)	Proportion CIs for all residents (%)	Residents who completed STEGH during residency (n thinsp;= 33)	Proportion CIs for residents completed STEGH during Residency (%)	Residents who did not participate in STEGH during residency (n = 88)	Proportion CIs for Residents who did not Participate in STEGH During Residency (%)	Chi-square
**Gender (%)**							
Female	57 (45)	36-54	15 (44)	29-61	42 (48)	38-58	.823
Male	64 (55)	46-64	18 (55)	38-71	46 (52)	42-62	
**Global health experience prior to residency (%)**							
Yes	78 (67)	58-75	27 (81)	65-91	51 (58)	48-68	0.029
No	39 (33)	25-42	6 (19)	9-35	33 (42)	32-52	
**Global health elective during residency (%)**							
Yes	33 (28)	21-37					
No	84 (72)	63-79					
**Location of experience (%)**	Prior to residency		During residency				
Africa	11(14)	8.9-21	13(39)	24-56			
Asia	7(9)	5-16	5(15)	6.6-31			
Americas	28(36)	28-45	7(21)	11-38			
Oceania	0	n/a	2(6)	1.7-20			
Two or more*	32(41)	33-50	6(18)	8.5-34			
Type of setting (%) **							
Rural			17(51)	35-67			
Suburban			2(6)	1.7-20			
Urban			14(42)	27-59			
**Level of training of respondent (%)**							
PGY-1	13(11)						
PGY-2	24(21)						
PGY-3	44(38)						
PGY-4	35(30)						

*Two or more distinct experiences across continents.

**Type of setting of longest experience.

After the survey was completed, residency coordinators were contacted and they provided all existing milestone data from each survey respondent in a de-identified format using a code that the respondent had created when taking the survey. The survey was initially piloted to graduating residents from the 4 institutions at which IRB exemption had been obtained. After the pilot was complete, the survey was sent to 641 residents in 15 programs. The programs were selected due to having a global health faculty member recruited via a request for assistance to members of the Global Emergency Medicine Academy of SAEM (https://www.saem.org/gema), who was willing to assist with survey distribution and facilitating the request for milestone data from residency leadership. Two residency programs did not provide milestone data from their residents. In total, individual surveys were gathered from 117 EM residents from 13 nationally accredited U.S. programs, and we were able to obtain milestone data on 80 of these residents. Of the residents for whom we received a set of milestone data, 46 residents had a complete set of milestone data from their residency training, while the remainder were still in the process of completing their training programs and only had a partial set of data. All milestone data was anonymized and linked to the survey.

The ACGME requires each accredited EM training program to evaluate each resident twice annually. Milestone data was thus available for residents at each of the milestone assessments conducted at 6-month intervals in years 1-3 or 1-4 (depending on length of the residency training program). There are six competency domains: patient care, medical knowledge, systems-based practice, practice-based learning and improvement, professionalism, and interpersonal and communication skills, with milestones assessed in 23 separate items across the six domains. Scores for each item range from 1 to 5, with increments of 0.5 between levels as the smallest possible significant difference between scores.^10^ The milestone domains were summed within each competency to provide an overall score which represents milestone achievement over time for each group.

Analysis and outcome measures: For analysis, EM residents were divided into two groups - those who had STEGH (pre-residency, during residency, or both) and those who had not. Chi-squared analysis and t-test were conducted to assess univariate difference between GH and non-GH experienced residents, a generalized linear model (GLM) assessed the effects of time and GH experience with interaction on each of the six competencies. Statistical analyses were conducted using SAS Version 9.4. Residents contributed varying amounts of competency data based on their progress in their residency training (first 6-month assessment to assessment for all 4 years of residency). This was accommodated in the regression model.

Funding sources: None.

Type of consent used: Electronic written consent was obtained from individual subjects as part of the online survey.

Access to underlying research materials: Deidentified data is stored electronically in the cloud and can be viewed by request.

## Results

The response rates of the pilot survey and the general survey of residents were 34% and 18.5%, respectively. Among 117 EM residents who completed the survey, 57 (45%) were female, the mean age was 30 years (standard deviation = 3.1), the majority (68%) were in their PGY3/PGY4 year, and 84 (71.8%) reported STEGH. 78 (67%) had STEGH prior to residency, and 33 (28%) had STEGH during residency (Table 2). Not all pre-residency experiences were short term in nature, as 13 residents had experiences lasting one year or longer prior to starting residency. More information on the duration of experiences pre-residency (during residency the majority of experiences were 3-5 weeks in length), primary role, and type of institution is provided in Table 3. The most common regions where experiences occurred pre-residency were in the Americas (36%), although many respondents (41%) had more than one experience. During residency most STEGH were done in Africa (39%). A total of 51% of experiences were in a rural setting. Chi-squared testing showed that the residents who had a STEGH during residency were more likely to have had previous STEGH than the residents who did not have a STEGH during residency (*P* = .03).

**Table 3.** Characteristics of STEGH participation By survey respondents.

**Duration of Experience**	Pre-Residency
≤ 2 Weeks	4 (5%)
2-4 Weeks	27 (33%)
5-12 Weeks	24 (29%)
13-52 Weeks	14 (17%)
≥ 52 Weeks	13 (16%)
**Role During Pre-Residency Experience**	
Clinical (Shadowing)	44
Clinical (Hands-on)	56
Clinical Research	13
Public Health Work	44
Other (Peace Corps, ESL Teaching)	3
**Role During Residency Experience**	
Clinical Care (no attending supervision)	14
Clinical Care (with attending supervision)	21
Teaching Medical Knowledge/Skills	17
Public Health Promotion	9
Clinical Research	6
Other	2
**Type of Institution (Residency Experience)**	
Tertiary Care/Academic Center	9
Regional Hospital	8
District Hospital	12
Non-Hospital Setting (ie Clinic)	11
Other (EMS system)	1

**Figure 1.**
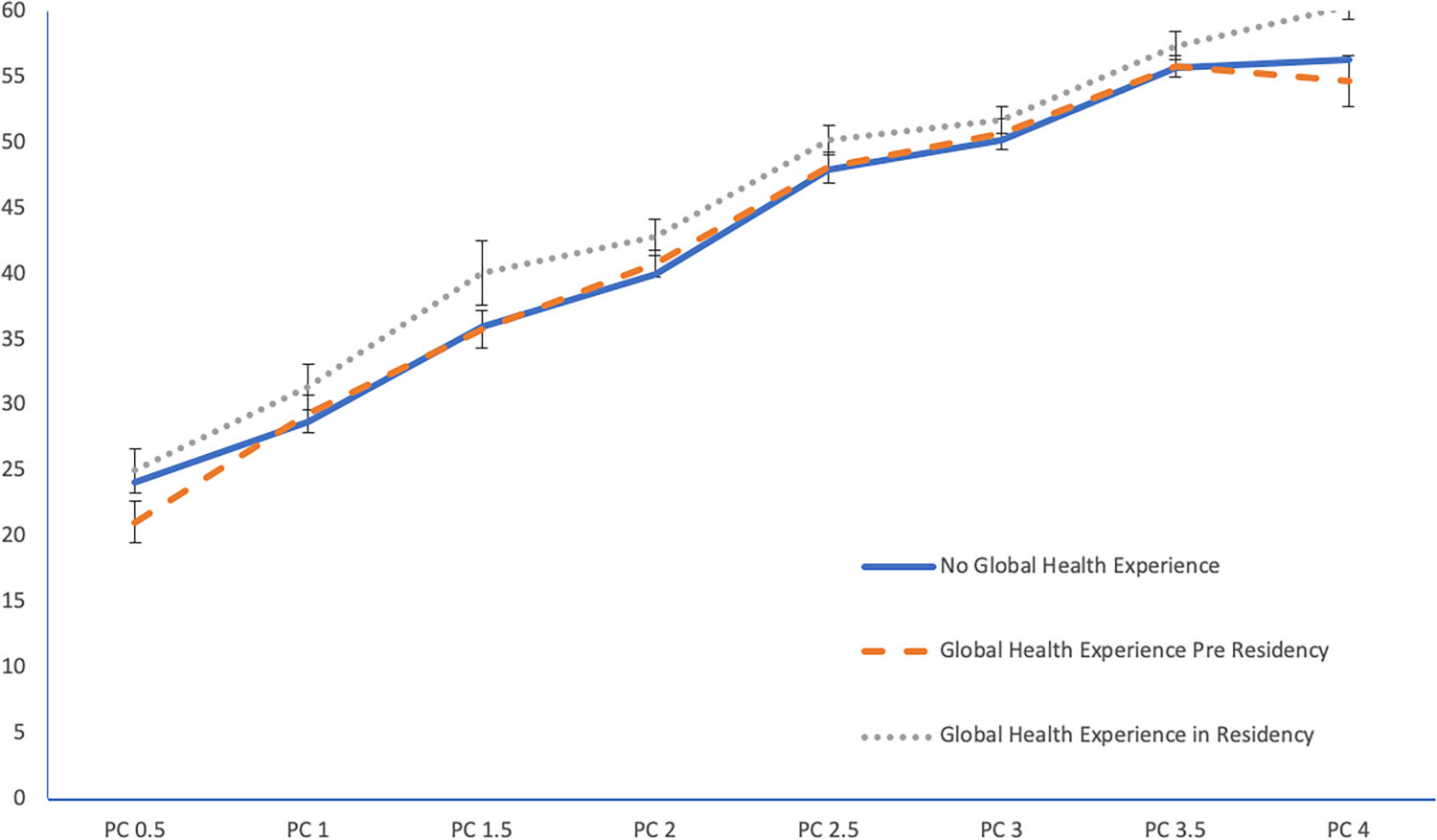
Change in mean patient care competency over time by global health experience.

Overall, residents received higher average milestone scores in all six competencies as they progressed through their residency training (*P* < .001). Per GLM analysis, the residents with any type of STEGH scored significantly higher than those without in the competencies of medical knowledge (*P* = .01), practice-based learning and improvement (*P* = .03), and professionalism (*P* = .01). There was no significant difference in scores between those with or without STEGH in patient care (*P* = .13), systems-based practice (*P* = .06), or interpersonal communication and skills (*P* = .12). There was no significant effect from the interaction between time and STEGH on milestone scores, suggesting that the rate of change in milestone scores over time were not different between those with or without GH experience. This means that residents with global health experience during their residency program performed better on these competencies than their counterparts without GHE, and that effect stayed constant over time. An illustration of the comparison between each group (pre-residency STEGH, STEGH during residency, and no experience) for the patient care competency is shown in Figure 1.

In a series of regression models, the effects of three levels of GH experience (no experience, experience solely pre-residency, and any previous global health experience including a residency elective) were examined with regard to change in milestone achievement over time (Table 4). All resident groups improved their performance during the duration of their residency training, but as can be seen in Table 4, across all competencies residents who undertook STEGH during the course of residency had significantly better performance (averaged over time) compared to either the group having experience before residency or the group that had no GH experience. There were no significant differences in performance between the GH experience prior to residency and no STEGH groups.

**Table 4.** Comparison of emergency medicine milestone score achievement by competency domain over time For residents With No global health experience, participation in STEGH before residency, And during residency.

Competency	No Global Health Experience^a^ n = 33 Mean overall performance (SD)	Mean CIs for No Global Health Experience	Global Health Experience pre- residency only^b^ n = 51 Mean overall performance (SD)	Mean CIs for Global Health Experience pre-residency only	Global Health Experience in Residency^c^ n = 33 Mean overall performance (SD)	Mean CIs for Global Health Experience in Residency	p-values and comparisons
Patient Care (PC). Items = 14	42.41(3.62)	41.13-43.69	42.13 (4.43)	40.88-43.38	44.92 (3.21)	43.78-46.06	A v B, *P* = .70 A v C, *P* = .004 B v C, *P* = .001
Medical Knowledge (MK). Items = 1	2.52 (0.40)	2.38-2.66	2.52 (0.50)	2.38-2.66	2.94 (0.34)	2.82-3.06	A v B, *P* = .97 A v C, *P* < .001 B v C, *P* < .001
Systems based practice (SBP). Items = 3	9.42 (0.75)	9.15-9.69	9.67 (0.93)	9.41-9.93	9.89 (0.63)	9.67-10.11	A v B, *P* = .16 A v C, *P* = .01 B v C, *P* = .21
Practice based learning and improvement (PBLI). Items = 1	2.92 (0.23)	2.84-3.00	2.94 (0.36)	2.84-3.04	3.14 (0.22)	3.06-3.22	A v B, *P* = .81 A v C, *P* = .001 B v C, *P* = .002
Professionalism (PROF). Items = 2	5.91 (0.52)	5.73-6.09	6.0 (0.64)	5.82-6.18	6.42 (0.50)	6.24-6.60	A v B, *P* = .53 A v C, *P* = .01 B v C, *P* = .02
Interpersonal and communication skills (ICS). Items = 2	6.16 (0.63)	5.94-6.38	6.17 (0.71)	5.97-6.37	6.53 (0.37)	6.40-6.66	A v B, *P* = .96 A v C, *P* = .01 B v C, *P* = .01

^a^ No Global Health Experience; ^b^Global Health Experience pre-residency only; ^c^STEGH in Residency.

## Discussion

This data demonstrates that STEGH among EM residents is associated with higher milestone scores in certain domains including professionalism, practice-based learning and improvement, and medical knowledge. Additionally, the completion of a STEGH during residency was associated with higher milestone scores as compared to residents with no STEGH or experience completed prior to residency. This suggests that STEGH experience during residency affords the resident greater competency to a measurable degree reflected in their milestone assessments.

Over the past few decades, healthcare professionals and trainees have become more aware of global disparities in health, and this has brought an unprecedented growth in academic GH programs. Many have focused their lens on the efficacy and sustainability of GH programs, resulting in the creation of long-term partnerships aiming to improve healthcare in lower-income countries.^16,17^ However, the effect of STEGH on trainee competency has been less well studied, and the studies largely have been qualitative in nature.^18–20^ To the best of our knowledge, our study is the first to analyze the relationship between ACGME milestones and STEGH exposure among EM residents. Other studies have explored subjective assessments of the relationship between STEGH and residency milestones through methods such as analysis of reflective writing assignments.^21,22^ Despite the dearth of relevant literature, we have attempted to contextualize the significant findings of our study within the literature by domain.

### Professionalism

Based upon review of our survey responses, which included qualitative descriptions from the residents of the reasons they felt their experiences might affect milestone achievement, we posit that experiences interacting with diverse cultures and under-resourced communities might result in development of competency in difficult to teach skills such as professionalism through cultivation of empathy, cultural competence, and humanism. Previous studies of healthcare professionals participating in STEGH have documented growth in empathy as well as cultural understanding.^23,24^

### Practice-Based Learning and Improvement

The ACGME describes competency in Practice-Based Learning and Improvement as having the ability to investigate and evaluate patient care practices, appraise and assimilate scientific evidence, and improve the practice of medicine.^10^ Most previous studies on the subject of the impacts of STEGH in residency do not assess this competency, likely because these studies have historically been self-assessments by residents only. However, a number of studies note that residents participating in STEGH spent significant time during the experience trying to evaluate and improve the patient care in which they were involved, accessing any available educational resources to do so, or improvement in “resourcefulness”.^13,24–26^ Residents used to practicing with supervising attendings always immediately at hand may have felt more motivated to appraise the practice of medicine when in a less closely supervised setting (as noted in Table 3, a number of residents reported no attending supervision during their STEGH, and some noted limited availability of attending physicians in free text responses).

### Medical Knowledge

Having the opportunity to practice medicine with limited availability of laboratory testing and radiologic studies may encourage increased development of medical knowledge of clinical skills such as physical diagnosis, while exposure to pathology that differs from that in the location where a resident's training program is located (such as tropical infectious diseases) also offers the opportunity to broaden one's medical knowledge base.^11,13,20,24–28^ Residents participating in STEGH in resource limited settings must learn new ways of using, reusing, or stretching the resources that are available to them, and creates an environment that encourages residents to seek evidence for their medical practice to ensure appropriate resource utilization, thereby presenting opportunities to actively pursue practice-based learning and improvement. Further research is needed to clarify the etiology of these effects.

### Domains Without Significant Effect

Other studies have found that healthcare workers participating in STEGH report significant impacts on communication skills, team building and leadership^23^ – our analysis does not show a significant impact on interpersonal and communications skills (ICS) competency as perceived by the residency Clinical Competency Committee (CCC) when looking at the residents with STEGH as a group, however when broken into subgroups, there appeared to be a significant effect of STEGH during residency in all competency areas. These results should be interpreted with caution, however, given the small numbers of residents in each group.

Despite increasing resident participation in GH efforts around the world, it has been theorized that diverting our institutional and personal resources for international training programs is not cost-effective, and there has been little research done on the potential effects of such experiences on resident education. Trainees participating in STEGH often do not have access to the level of technology and systemic support that they do in the U.S., and concerns have been raised that such an environment may not be suited for optimal trainee education ^20,27,28^ Our findings suggest that STEGH are positively correlated with trainee performance in certain domains of ACGME competency, and that STEGH during residency may be associated with improved performance in all domains. The lack of a negative correlation between STEGH and resident competency may help to alleviate concerns that these types of electives could hinder trainee performance.

## Limitations

Our study was limited by the challenges of obtaining full milestone data from residency programs, particularly given that it required additional work for residency coordinators. Another issue was a low response rate to our survey, which may have been related to residents not understanding that they were eligible to complete it whether they had STEGH or not, as well as the difficulty in following up with residents who were at programs outside our institutions.

One way to draw causal inference is to compare the changes in milestone scores before and after the STEGH during residency. Unfortunately, due to the limitations noted, making a consistent comparison of the milestone scores before and after STEGH during residency was difficult. The study team often did not have access to the complete ACGME milestone scores for residents as the scores were obtained from residency coordinators who were sometimes unable to provide data from certain time periods. In addition, the precise timing of STEGH was not available for many residents to run the analysis with statistical precision. Milestone scoring standards can also vary among different residency programs. Certain residency programs may also have more residents with STEGH than other programs, which may ultimately have biased our results.

While we found that STEGH overall were positively associated with milestone scores from certain competency domains, we cannot determine if residents with STEGH actually benefited from their experiences per se or simply have differing qualities compared to those without the experience. For example, struggling residents are often prohibited from STEGH participation, and exemplary residents may participate in a wider array of extracurricular activities.

Finally, although our findings demonstrated statistically significant differences in milestone scores between residents with and without STEGH, it is not clear whether these differences are clinically or otherwise significant to their performance in residency programs and whether these effects are long-lasting.

## Conclusions

STEGH among EM residents was associated with higher ACGME milestone scores within the competencies of medical knowledge, practice-based learning and improvement, and professionalism. Completing a STEGH during residency had a greater impact than GHE prior to residency training, with positive effect suggested in all competency domains. These findings suggest that STEGH may have value for residents in EM training. Based on this data, we believe STEGH should be supported by graduate medical education institutions and departmental leadership. Further studies could elucidate whether such experiences have an impact on outcomes or other performance measures in residency training, such as in-service training exam or board score performance, or practice type and location post-residency.
